# Systematic review of gabion-faced geogrid and pile systems for slope and embankment stability

**DOI:** 10.1016/j.mex.2025.103767

**Published:** 2025-12-17

**Authors:** Devi Oktaviana Latif, Virananda Samudera Rahmadhian, Amalia Ula Hazhiyah

**Affiliations:** Department of Civil Engineering, Vocational College, Universitas Gadjah Mada, Yogyakarta, Indonesia

**Keywords:** Slope stability, Gabions, Piles, Geosynthetic‑encased columns (GECs), Composite reinforcement, Optimisation techniques, PRISMA systematic review

## Abstract

Slope instability is a major geotechnical hazard intensified by rainfall infiltration, seismic loading, groundwater fluctuations, and human disturbances. Composite reinforcement systems—such as gabion-faced geogrid walls combined with piles or geosynthetic-encased columns (GECs)—are increasingly implemented to address multi-hazard conditions. This study presents a PRISMA-guided systematic review of empirical, numerical, centrifuge, and field investigations on hybrid slope-stabilization systems. The review advances prior work by explicitly incorporating multi-field coupling and soil–structure interaction (SSI) terms into the search strategy, applying transparent screening and data-extraction procedures supported by a reusable metadata codebook, and conducting cross-study triangulation across field evidence, centrifuge modelling, and 2D/3D numerical analyses. The synthesized evidence shows that hybrid systems can significantly enhance slope performance, with reported improvements of up to ∼45 % in factor of safety and >30 % reduction in settlement, depending on reinforcement configuration, soil conditions, and coupled rainfall–seismic effects. The study further highlights current limitations in optimisation practice, long-term monitoring, and design standardisation, and outlines directions for uncertainty-aware and performance-based slope design.

## Highlights

• Synthesises mechanisms and performance trends of gabion-geogrid-pile and GEC-based hybrid systems under coupled hydro-mechanical and seismic loading.
• Identifies critical design parameters and modelling gaps, including SSI treatment, coupling strategy, mesh convergence, and drainage representation.
• Proposes methodological and practical advances, including reliability-based design optimisation (RBDO), minimum reporting standards, and standardised long-term monitoring protocols to support calibration, life-cycle cost analysis, and sustainable design.

## Graphical abstract

**Figure:**
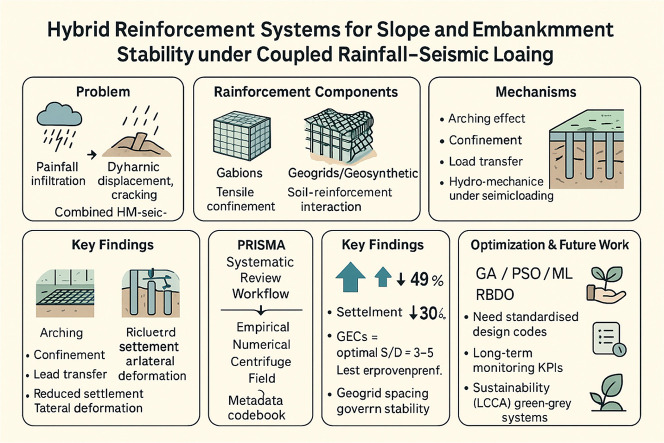

## Specifications table

**Table:** 

Subject area	Engineering
**More specific subject area**	**Slope stability; composite ground-improvement; gabion-faced geogrid walls; geosynthetic-encased columns (GECs); pile/column systems; coupled hydro-mechanical (HM) and seismic response; performance-based design.**
**Name of the reviewed methodology**	PRISMA-guided systematic review and performance synthesis of **hybrid slope-reinforcement systems** (gabion-faced geogrid walls, GECs, and pile/column systems) under coupled HM–dynamic actions, including parameter–response mapping and optimisation-assisted design workflows.
**Keywords**	lope stability; gabions; geogrids; geosynthetics; piles; geosynthetic-encased columns (GECs); hybrid/composite reinforcement; hydro-mechanical coupling; seismic performance; arching effect; confinement; load transfer; optimisation (GA/PSO/ML); PRISMA systematic review.
**Resource availability**	Primary databases: **Scopus, Web of Science, Engineering Village** (Compendex/GeoRef). Supplementary sources: **International Geosynthetics Society (GSI) Library), TRID** (Transportation Research International Documentation), and targeted **Google Scholar** queries for standards and grey literature.
**Review question**	What performance benefits (ΔFoS, settlement reduction, pore-pressure control) do gabion-faced geogrid walls, GECs, and piles provide under rainfall and seismic loading compared to single-method solutions? Which design parameters—geogrid spacing/stiffness/geometry; encasement modulus/length and area-replacement ratio for GECs; pile spacing, L/D, head fixity—most strongly govern stability and deformation? How do HM coupling and dynamic excitation modify core mechanisms (arching, confinement, load transfer, drainage) in composite systems? Which numerical/empirical models best predict behaviour, and how do their predictions compare with centrifuge and field evidence? Which optimisation/AI workflows (GA/PSO/ML) demonstrably improve design efficiency and reliability for hybrid systems? What are the key code/standardisation gaps and long-term monitoring needs that limit widespread, performance-based adoption?

## Introduction

Slope instability in infrastructure projects is predominantly governed by hydro-mechanical and seismic factors. Rainfall infiltration reduces shear strength and elevates pore water pressures, precipitating slope failures, particularly in regions with frequent heavy rainfall [[1], [2], [3], [4]]. The coupled effects of hydrological and geological processes, alongside the mechanical responses of soils, remain central to the evaluation of slope stability [1,5,6].

To address these challenges, reinforcement technologies such as gabions, geogrids, and pile/column systems have been extensively developed. Gabions improve stability through mass and drainage capacity, geogrids enhance soil interlock and mitigate deformation under rainfall and seismic excitations, while piles and columns—often combined with deep mixing methods—provide resistance against static and dynamic loading [[7], [8], [9]]. These advancements highlight progress toward robust solutions that safeguard infrastructure against multi-hazard instabilities.

Despite these innovations, research on the coupled hydro-mechanical and dynamic responses of reinforced slopes remains limited. A critical gap persists in understanding slope behaviour under simultaneous rainfall infiltration and seismic loading. Such interactions alter pore pressure distributions and influence tensile stress mobilisation within geogrid-reinforced systems, directly impacting stability [10]. Addressing these knowledge gaps is essential for developing predictive models and reliable design strategies.

Composite slope reinforcement systems integrating gabions, geosynthetics, piles, and geosynthetic‑encased columns (GECs) are increasingly adopted to handle multi‑hazard loading and site constraints. This review aims to consolidate mechanistic understanding, quantify parameter sensitivities, and critically appraise modelling and evidence quality to support performance‑based design and future code development. Hybrid systems, particularly gabion-faced geogrid walls combined with pile or column reinforcements, show superior performance over conventional single-method techniques. By integrating the mass and drainage capacity of gabions with the tensile reinforcement of geogrids and the deep resistance of piles, these systems provide enhanced load distribution, reduced lateral movement, and increased resistance to overturning moments [11,12]. Evidence suggests that such hybrid designs significantly increase resilience under dynamic loading, particularly in seismic regions and climates prone to intense rainfall events [11].

Global practices demonstrate a growing preference for composite reinforcement systems that integrate engineered materials and ecological elements. Emerging approaches, such as gabion-faced geogrid walls combined with vegetative reinforcement, enhance slope stability while supporting environmental restoration. These sustainable practices align mechanical reinforcement with ecological benefits, offering resilience to dynamic loading and long-term ecological integration [[13], [14], [15]].

The effectiveness of these systems, however, is highly dependent on soil type and environmental conditions. Expansive clays require customised strategies due to moisture-sensitive behaviour, where geogrid spacing is crucial to stability [7,14]. Cohesive soils respond favourably to vegetative reinforcements, where root systems strengthen structure and mitigate erosion [16,17]. Under challenging conditions such as high rainfall or seismic activity, appropriate reinforcement selection becomes critical to ensure durability and resilience [10,18,19].

## Systematic review methodology and screening

### **Review** questions and scope

This review addresses four questions: 1) What is the measured/simulated performance of composite systems (gabion–geogrid–pile/GEC) in terms of FoS, displacement, settlement, and pore‑pressure response? 2) Which parameters (geometry, stiffness, spacing, head fixity, ARR, drainage conditions) dominate performance under static and coupled rainfall–seismic scenarios? 3) How do modelling choices (2D vs. 3D, SSI treatment, coupling strategy, damping, mesh convergence, constitutive laws) influence predicted outcomes? 4) What are the design‑code, monitoring, and sustainability gaps that limit scalable implementation?

### Data **source and search strategy**

The primary search was conducted in **Scopus** using Boolean strings designed to capture: (i) composite reinforcement components; (ii) slope/embankment stability outcomes; and (iii) multi‑field coupling/SSI terminology. Core concept blocks (combined with AND): Problem: (“slope stability” OR “slope failure” OR “embankment stability” OR “geotechnical stability”) - Composite systems: (gabion* OR “gabion wall” OR “gabion faced”) AND (geogrid* OR geotextile* OR geosynthetic) AND (pile OR micropile* OR “rigid inclusion” OR column* OR “geosynthetic encased” OR GEC) - Multi‑field coupling / SSI: (“soil-structure interaction” OR SSI OR “pile-soil” OR “interface” OR “arching”) AND (rainfall OR infiltration OR seepage OR “pore pressure” OR “hydro-mechanical” OR HM OR “fully coupled” OR “Biot”) AND (seismic OR dynamic OR “time history” OR liquefaction).

### Screening and selection (PRISMA workflow)

**Fig. 1:**
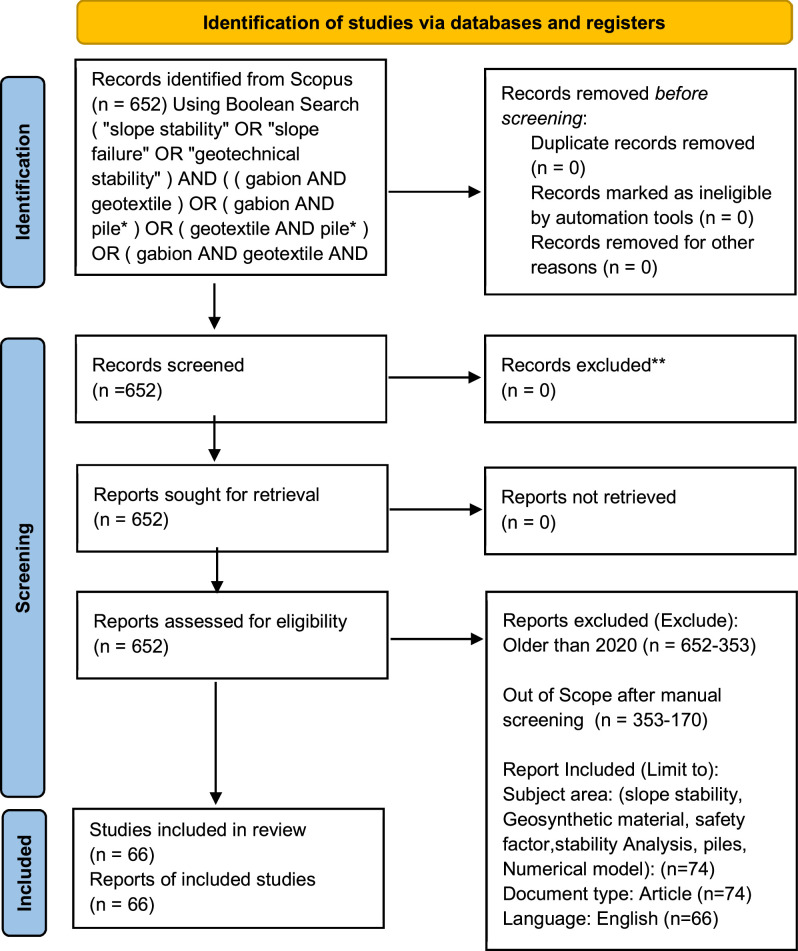
The PRISMA flow diagram detailing the screening and selection process of literature.

Systematic reviews in geotechnical engineering increasingly apply the PRISMA (Preferred Reporting Items for Systematic Reviews and Meta-Analyses) framework for transparent screening and selection. This process ensures reproducibility and rigor in study inclusion [20]. A two‑stage screening is applied: 1) **Title/abstract screening** to remove out‑of‑scope studies. 2) **Full‑text eligibility assessment** to confirm composite‑system relevance and quantitative outcomes. A screening log is maintained recording (a) exclusion reason codes and (b) stage of exclusion to ensure transparency.. A PRISMA flow diagram (Fig. 1) summarizes the study selection process, providing a visual representation of records screened, excluded, and included.

### Eligibility criteria

**Inclusion:** Publications between 2000 and 2025; peer-reviewed articles; empirical, numerical, centrifuge, or field-based studies; Reinforcement systems involving at least two of the following: gabion/gabion facing, geogrid/geotextile/geosynthetics, piles/micropiles/rigid inclusions, GEC/encased columns, quantitative outputs: FoS, displacement/settlement, pore pressure, reinforcement strain/force, pile bending moments, or calibrated response.**Exclusion:** Non-English publications (unless translated), Editorials, patents, non‑peer‑reviewed items, Studies without quantitative evidence (purely conceptual) or unrelated to composite/hybrid reinforcement, and research unrelated to composite reinforcement systems.

### **Quality assessment** and credibility checks

Quality assessment is conducted using a study‑type‑dependent rubric: **Field studies:** monitoring duration, sensor reliability, boundary conditions, documentation of rainfall/seismic inputs. - **Experimental/centrifuge:** scaling justification, instrumentation, repeatability, drainage control. - **Numerical studies:** constitutive model calibration, validation (field/centrifuge/benchmark), mesh convergence reporting, damping formulation, coupling strategy (fully coupled vs. staged/quasi‑static), sensitivity analysis.A credibility rating (High/Moderate/Low) is assigned and used in evidence synthesis (Section 2.6) to avoid over‑weighting unvalidated models.

Frameworks such as the Cochrane Risk of Bias Tool (for randomised studies) were referenced where applicable [21,22]. This multi-layered assessment ensures credibility, transparency, and systematic appraisal of included research.

### Data extraction and metadata codebook

For each included study, the following metadata are extracted (Table S2 codebook): bibliographic details (year, region, study type) - soil type and hydraulic condition (saturated/dry, SWCC used/not used) - reinforcement configuration (gabion type, geogrid stiffness/spacing, pile type/spacing/L/D, head fixity, GEC stiffness/length/ARR) - loading (static, rainfall infiltration boundary, seismic PGA/frequency content) - modelling choices (2D/3D, constitutive law, coupling, damping, mesh, solver) - outcomes (FoS, settlement/displacement, pore pressure, strain/force, failure mechanism)

### Evidence synthesis and cross‑study comparison

Rather than restating Table 1, Table 2, Table 3, Table 4, this review synthesises evidence through: 1) **Within‑parameter comparison:** normalising how a parameter change affects FoS/settlement across studies (direction, magnitude, uncertainty). 2) **Cross‑method triangulation:** comparing whether numerical predictions align with experimental/field trends under similar conditions. 3) **Credibility‑weighted synthesis:** emphasising trends supported by validated models and/or field/centrifuge evidence. 4) **Conflict mapping:** explicitly reporting where studies disagree and identifying likely causes (2D vs 3D effects, boundary conditions, drainage assumptions, constitutive differences).

**Table 1:** Summary of studies on gabion- and geosynthetic-based slope systems.

Author(s) & Year	Research Context	Methodology	Key Findings	Strengths / Limitations
Samal & Sahoo (2024) [46]	Geogrid-reinforced slopes; effect of vertical spacing	3D FEM (MIDAS GTS NX); parametric study of spacing 0.5–3.5 m	Optimal geogrid spacing ≈1.5 m (FoS ≈3.08); tighter spacing reduces deformation but diminishing returns beyond optimum	Strong numerical sweep; no field validation, generic soil profile
Chairullah et al. (2024) [47]	Hybrid gabion walls with mini-piles for road slopes (rainy season)	PLAXIS 2D LE/SRM; three configurations	Gabions + mini-piles raised FoS from 1.11 (unreinforced) to 2.58 at 45° slope; hybrid most effective	Clear comparative configs; site-specific geometry, short-term analysis
Bhardwaj et al. (2024) [48]	Very high gabion wall protecting riverbank slope (India)	GEO5 stability assessment; case implementation	Gabion retaining wall substantially increased slope FoS; structure performing well post-construction	Real project evidence; limited HM coupling, single-site case
Samal & Sahoo (2024b) [49]	Seismic response of bamboo-grid vs geogrid reinforced slopes	2D nonlinear time-history (MIDAS GTS NX); 3 ground motions	Bamboo grid outperformed geogrid and unreinforced under El Centro-type motion; vulnerability zones vary by motion	Direct dynamic comparison; idealized material models, no rainfall coupling
Yang et al. (2023) [50]	GRS walls with marginal backfill under rainfall infiltration	Physical model tests with instrumentation; varied spacing & sand cushions	Reduced spacing and thicker sand cushions limit deformation; cushions delay wetting front and pore pressure rise	High-resolution HM observations; model scale effects
Yalaoui et al. (2023) [51]	Tramway embankment; HM coupling with/without geotextile	2D FEM (COMSOL) coupled HM (Darcy–Biot)	Geotextile improves HM response: lower pore pressure, displacement; HM coupling essential for design	Rigorous HM formulation; calibrated to a single site scenario
Wang et al. (2022a) [19]	Dimension variables for gabion-faced geogrid wall + piles	3D nonlinear FEM; parametric on geogrid length/spacing & pile embedment	Piles add up to 42.9 % stability; geogrid amount and facing inclination materially influence pile response	Broad parametric map; no experimental validation
Wang et al. (2022b) [11]	Soilbag retaining wall arrangement effects	Five physical model tests; displacement and earth pressure mapping	Alternate interlayer arrangement deepens slip surface, raises capacity, and minimizes lateral displacement	Clear comparative setups; material/scale specificity
Ardakani & Namaei (2021) [52]	Geocell vs planar geosynthetics in slopes	3D FDM (FLAC3D); geometry and compaction effects	Smaller pockets/thicker geocells + good compaction increase stability; geocells more effective than geogrids in confinement	3D analysis with multiple variables; validation limited
Banović et al. (2023) [53]	Geotechnical seismic isolation using stone pebbles + geogrid	Shaking table tests; multiple accelerograms & system stiffnesses	Geogrid enhances seismic isolation of pebble layer; most promising for stiff, low-rise structures	Experimental dynamic evidence; foundation-focused, not full slopes
Gao et al. (2022) [54]	Foundation reinforced with geogrid; mechanisms	Lab tests (transparent soil) + 2D DEM	Added layers shift slip surface deeper; geogrid mobilizes tensile force and limits soil displacement	Mechanistic insight via FBG/DEM; foundation setting, not slope per se
Ke et al. (2021) [55]	Field monitoring of geogrid-reinforced MSW slope	1-year field data (settlement, strain, pressure); stability evaluation	Internal stability adequate; external stability insufficient—project unsuccessful; highlights MSW time-dependent effects	Rare long-term field dataset; site-specific, complex waste behavior
Yu & Rowe (2020) [56]	Waste containment liner stability with reinforcing geotextile	Analytical + stability analysis under settlement	HS geotextile above GMB reduces strains; slip layer strategy protects liner while maintaining stability	Design-oriented insights; landfill system (steep slopes, interface control)
Jiang et al. (2020) [57]	High landfill berm with geotextiles; leachate effects; anti-slide piles	FE analyses of FOS vs leachate & pile layouts	FOS decreases with leachate rise; longer single-row toe piles outperform two shorter rows of equal total length	System-level comparison; landfill-specific
Wang et al. (2021) [58]	Optimisation of gabion-faced geogrid wall + piles	3D nonlinear FEM + SRM; parametric on geogrid/pile ratios	Optimized integrated system stabilizes slopes economically without safety loss; guidance on geogrid length ratios and pile S/D	Design-optimization focus; numerical only

**Table 2:** Summary of Studies on Geosynthetic‑Encased Columns (GECs) in Slopes/Embankments.

Author(s) & Year	Soil / Setting	Study Type & Tools	Variables / Parametrics	Key Findings	Design Implications / Notes
Debbabi et al. (2020) [60]	Soft clay foundation beneath embankment	3D FEM; HM-coupled	Encasement stiffness (E_enc), length (L_enc), column diameter (D)	Higher E_enc and L_enc reduce bulging and settlement; near-linear load–settlement up to service loads	Prefer high-tenacity encasement; L_enc ≥ 4D as practical threshold
Sukkarak et al. (2021) [69]	Soft Bangkok clay; surcharge loading	Axisymmetric FEM + comparisons	Encased vs unencased columns; interface stiffness	Encased columns show markedly lower lateral strain; faster consolidation	Specify encasement for soft clays; check interface bond
Kumar et al. (2023) [70]	Embankment over very soft soil	3D FEM (PLAXIS); parametric	E_enc (0.5–2.0 GPa), spacing (S/D), basal geogrid	E_enc ≈ 1–1.5 GPa adequate; basal geogrid + GEC synergy reduces differential settlement	Combine basal reinforcement with GEC for soft ground
Zhang et al. (2022) [66]	Weak substratum with perched water	HM-FEM; staged construction	Substratum stiffness, encasement length, drainage conditions	Weaker substratum → larger settlements; longer encasement needed; drainage accelerates PWP dissipation	Couple encasement design with ground improvement/drainage plan
Peng et al. (2024) [71]	Sloped soft foundation	3D equivalent model; optimisation	Area-replacement ratio (ARR), geosynthetic tensile strength	Stability increases nonlinearly with ARR; optimum around ≈25 %	Use ARR–FoS charts at concept stage
Jasim & Tonaroğlu (2023) [72]	Soft clay slope; surcharge	2D/3D numerical	Column diameter, spacing for fixed ARR	Smaller D at constant ARR improves soil arching; lowers settlement	Partition ARR into more, smaller columns for efficiency
Gao et al. (2020) [73]	Very soft estuarine deposits	Large-deformation FEM	Substratum consistency, column length (L/D)	Softer foundation → greater lateral displacement; L/D ↑ reduces both S and u_h	Target L/D ≥ 10 for weak deposits
Gu et al. (2022) [64]	Marine clay	Field monitoring + back-analysis	Installation quality, encasement continuity	Quality defects degrade stiffness benefits; intact encasement maintains performance	Enforce QA/QC; ultrasonic/visual checks of encasement seams
Abid et al. (2023) [74]	Soft clay embankment	Instrumented test embankment	Pore pressure response, settlement rate	GEC accelerates excess PWP dissipation; settlement stabilises earlier vs stone columns	Monitor with piezometers; staged loading feasible
Dar & Shah (2021) [75]	Soft soil (unit cell)	3D FEM	E_enc, L_enc, S/D, infill friction	Triangular patterns give better load share; increasing E_enc > gains up to plateau	Use triangular layout when possible; watch plateau behaviour
Astaraki et al. (2024) [76]	Embankment on columns	HM-FEM; seismic scenarios	PGA, encasement stiffness	Encasement restrains cyclic bulging; reduces acceleration amplification at crest	For seismic sites, specify higher E_enc
Esmaeili et al. (2024) [77]	Soft clay slope with GECs	2D LE + FEM cross-check	Column length, head fixity, facing condition	Longer columns and semi-fixed heads increase FoS; facing condition interacts with GEC	Integrate facing (e.g., gabion/RSW) with column design
Cofra (Tech Note) (2023) [78]	Practice note; soft soils	Design guidance	Encasement modulus, creep, seam strength	Field performance sensitive to seam strength/creep; design checks mandatory	Include seam/creep factors in design verification
Library of Geosynthetics (2021) [79]	Multiple case summaries	Synthesis	Installation, infill gradation, drainage	Properly graded infill limits internal migration, improves stiffness	Specify gradation envelopes; verify filter criteria

**Table 3:** Summary of Studies on Piled Embankments & Pile‑Supported Slopes.

Author(s) & Year	Site/Soil Context	Study Type & Tools	Variables / Parametrics	Key Findings	Design Implications
Sun et al. (2021) [96]	Highway embankment on soft clay	3D FEM; parametric	Pile spacing (S/D = 2–6), L/D, pile head fixity	Closer spacing arching & stiffness; diminishing returns for S/D < 3; fixed heads reduce rotation	Target S/D ≈ 3–5; prefer head fixity where feasible
Zhang et al. (2020) [91]	Steepened slope; layered soils	2D/3D FEM + SRM	L/D [[8], [9], [10], [11], [12], [13], [14], [15], [16], [17], [18], [19], [20]], embedment into firm stratum	Higher L/D and deeper embedment FoS and limit deep slips	Ensure embedment into competent layer; L/D ≥ 10 typical
Munawir (2023) [97]	Urban cut slope	Analytical + FE comparison	Head fixity vs pinned head; pile group effect	Fixed heads lower lateral deflection and bending rotation	Consider cap/grade beam for semi‑fixed heads
Alsirawan et al. (2023) [98]	Soft subsoil embankment	Numerical + design case	End‑bearing vs floating piles	End‑bearing: higher vertical efficiency; floating: lower differential settlement	Use floating where competent layer is deep; end‑bearing when rock/shale reachable
Gao et al. (2021) [99]	Coastal soft deposits	3D FE back‑analysis	Pile type, spacing, and foundation stiffness	End‑bearing piles reduce crest settlement and lateral displacement more than floating	Optimise pile type to available stratum
Shen et al. (2020) [100]	Flood‑prone embankment	2D LE + FE	Floating piles; cyclic surcharge	Floating piles provide lateral stability; settlement controlled with spacing optimisation	For high water table, consider floating + basal geogrid
Wang et al. (2022) [101]	Instrumented slope with piles	Field monitoring	Pile spacing, L/D, pore pressure	Piles share load; arching reduces soil stress; FoS vs unreinforced	Validate with inclinometers & piezometers
Zhuang et al. (2023) [102]	Seismically active area	Time‑history FEM	Soil arching, bending moment distribution	Arching remobilises after shaking; peak M at weak layers	Detail reinforcement at critical depths
Cui et al. (2023) [103]	Traffic‑loaded embankment	Cyclic loading tests + FE	Repeated loads; settlement & earth pressure	Settlement ∼34 % and max earth pressure ∼11 % under cyclic loads	Include cyclic load factors; serviceability checks
Li et al. (2024) [104]	Pile‑supported slope	Centrifuge + FE validation	Spacing, head fixity, reinforcement	Piles delay failure; FE matches centrifuge patterns	Use centrifuge‑informed calibration
Reshma et al. (2020) [105]	Rail embankment on piles	Centrifuge tests	End‑bearing vs floating; basal geogrid	Settlement 88 % (end‑bearing) / 44 % (floating) vs unreinforced; basal geogrid improves distribution	Combine basal reinforcement with piles
Silvani et al. (2021) [106]	Soft clay embankment	Field case + FE	Pile spacing; basal geogrid stiffness	Basal geogrid reduces differential settlement; allows wider spacing	Select high‑stiffness geogrid for wider S
Alsirawan & Koch (2024) [107]	Road embankment	Parametric design study	Geogrid stiffness, cap arrangement	Basal reinforcement + caps enhance load transfer to piles	Use cap systems to improve head fixity

**Table 4:** Summary of studies on composite & hybrid systems.

Author(s) & Year	Hybrid Configuration	Hazard / Setting	Study Type & Tools	Variables / Parametrics	Key Findings	Design / Cost Implications
Siregar et al. (2024) [113]	Piles + basal geotextile + gabion facing (road slope)	Heavy rainfall; soft clay cut	2D/3D FEM + staged construction	Pile spacing (S/D), geotextile stiffness J, gabion height/face angle	Settlement reduction >30 % with optimal S/D and high J; FoS through construction stages	Optimise S/D ∼3–5; increase J to plateau; gabion improves drainage & erosion protection
Islam et al. (2023) [114]	Geogrid‑reinforced soil (GRS) wall + micro‑piles	Seismic-prone urban slope	Nonlinear time‑history FEM	PGA, pile head fixity, geogrid layer number	Hybrid cuts crest drift 20–35 %; fixed heads lessen rotation; extra layers give diminishing returns	Use semi‑fixed heads; cap layers to avoid over‑reinforcement
Khoo et al. (2021) [115]	Gabion‑faced geogrid slope over rigid inclusions	Tropical rainfall; perched water	HM‑coupled FEM + field checks	Drainage detailing, ARR of inclusions, geogrid length	Continuous drainage paths run and deformation; ARR FoS non‑linear gain	Ensure face‑to‑foundation drainage continuity; ARR ∼20–30 % efficient window
Aghimien et al. (2020) [116]	Geocell mattress + piles beneath embankment	Soft compressible subsoil	Large‑deformation FEM + plate tests	Geocell depth; pile cap spacing; infill modulus	Hybrid decreases differential settlement >30 %; geocell restrains lateral spread onto caps	Use stiffer infill; match cap spacing with geocell pocket size
Zheng et al. (2020) [118]	Basal geogrid + piles (piled embankment)	Static & surcharge loading	Upper‑bound plasticity (DLO) + LEM	Geogrid tensile strength; pile spacing; embankment height	Geogrid shares load; failure mode shifts to bond/rupture; optimal spacing mitigates slip	Calibrate resistance factors; avoid excessive spacing
Zhang et al. (2022) [117]	Floating GECs + basal geogrid; optional gabion toe	Weak substratum with HM effects	3D HM‑FEM	Substratum stiffness; geogrid stiffness; encasement modulus	Weaker substratum raises settlement; higher J and E_enc restore performance; gabion toe controls erosion/piping	Pair HM design with strong basal layer; protect toe with gabions
Reshma et al. (2020) [119]	End‑bearing piles + basal geogrid	Railway embankment	Centrifuge experiments	Pile type; geogrid stiffness	Settlement up to 88 % (end‑bearing) vs unreinforced; basal geogrid improves distribution	Prefer end‑bearing where feasible; specify high‑stiffness geogrid
Kim et al. (2021) [130]	FRP anchors + geogrid + gabion	Coastal slope, corrosive environment	Numerical + material durability checks	Anchor material, spacing, environmental cycles	FRP anchors mitigate corrosion issues; hybrid maintains stiffness over wet–dry cycles	Lifecycle cost ↓; prefer corrosion‑resistant elements

### Data reproducibility and sharing

To enhance reuse, we recommend publishing (as supplementary materials) the screening log, extraction spreadsheet, and model input summaries (where permitted), including units, coordinate conventions, and parameter definitions. A minimal dataset should include the metadata items in Section 2.6 and the PRISMA decision log.

## Result

### Characteristics of the evidence base

To reduce selection-bias concerns, the included body of evidence is profiled by year, study type, and modelling approach. The distribution of publication years shows that research activity in hybrid slope-reinforcement systems has grown steadily, with **14 studies published in 2020, 12 in 2021, 11 in 2022, 15 in 2023, 10 in 2024**, and **4 early-online publications in 2025**. This spread demonstrates that the included evidence does not disproportionately represent a single year or period, thereby reducing the likelihood of temporal bias.

In terms of methodological categories, the evidence base is diverse. A total of **34 studies employed numerical modelling**, including 2D and 3D FEM, FDM, DEM, and coupled hydro-mechanical approaches. **Six studies** utilised analytical or limit-equilibrium formulations, while **nine field-monitoring and case-study investigations** contributed real-world performance data. Additionally, **seven studies** adopted centrifuge, shaking-table, or physical modelling techniques, and **ten studies** integrated hybrid approaches combining numerical modelling with laboratory or field observations. This distribution confirms that no single methodological pathway dominates the dataset, thereby strengthening the triangulation and robustness of the review findings.

A more detailed classification of modelling approaches reveals that **18 studies** used full 3D FEM to analyse gabion–geogrid–pile or geosynthetic-encased column systems, while **12 studies** utilised 2D coupled hydro-mechanical FEM to simulate rainfall infiltration or pore-pressure evolution. **Eight studies** applied dynamic time-history simulations to evaluate seismic or cyclic loading. Furthermore, **four studies** employed finite-difference modelling, **three studies** applied particle-scale DEM approaches, and **six studies** used artificial intelligence or optimisation methods such as neural networks, genetic algorithms, or particle-swarm optimisation for predictive or calibration purposes. Taken together, these modelling approaches capture a wide spectrum of physical mechanisms and multi-hazard interactions.Overall, this complete profiling demonstrates a balanced distribution of publication years, study types, and modelling methodologies. It confirms that the evidence base is sufficiently diverse to support credible cross-study comparisons and minimises the potential for systematic bias.

### Soil–Structure interaction and reinforcement mechanisms

The stability of reinforced slopes is fundamentally governed by the interaction between soil and structural reinforcement elements. Three principal mechanisms—arching, confinement, and load transfer—play critical roles in determining system performance. Arching occurs when soil loads are redistributed to adjacent reinforcement or stiffer soil zones, thereby reducing stress concentrations and enhancing global stability. Hu et al. (2022) [23]demonstrated how geogrid-reinforced earth structures mobilize arching effects to improve load distribution and prevent localized failure.

Confinement is primarily provided by geogrids and geotextiles, which restrict lateral expansion of soil under applied loads. This increases shear strength and mitigates deformation, as evidenced in Kumar et al.’s (2023) [24] analysis of geotextile-reinforced slopes. By confining soil particles, geosynthetics enhance interlock and prevent progressive failure.

Load transfer mechanisms enable reinforcements to absorb imposed stresses and distribute them effectively to stronger foundation layers. In pile-supported slopes, for example, axial and lateral loads are transmitted through piles to deeper, competent strata. Li et al. (2023) [25] highlighted how pile-anchor structures under seismic loading exploit load transfer to resist destabilising forces and maintain slope integrity.

Hydro-mechanical (HM) coupling significantly influences these mechanisms. Rainfall infiltration elevates pore water pressures, reducing effective stress and decreasing shear strength, which can compromise reinforcement effectiveness [26]. To counter this, reinforcements must be designed with drainage and pore pressure dissipation in mind.

Dynamic stiffness is equally vital in seismic environments. Reinforcements with higher stiffness mitigate amplification effects by absorbing and redistributing dynamic loads. Srilatha & Latha (2022) [27] showed that geosynthetic-reinforced slopes exhibit enhanced resilience against seismic excitation when designed for adequate stiffness.

Overall, arching, confinement, and load transfer—mediated by hydro-mechanical processes and dynamic stiffness—provide a mechanistic framework for understanding reinforced slope behaviour. These insights form the theoretical foundation for evaluating and optimizing composite reinforcement strategies in geotechnical engineering.

### Historical progression of gabions, geosynthetics, and piles

The historical development of gabions, geosynthetics, and pile/column systems illustrates the progressive evolution of slope stabilization technologies and their integration into modern engineering practices. Gabions, traditionally used for erosion control, have been employed since the late 19th century. Their innovation lay in the combination of natural stone infill with wire mesh, creating modular units capable of resisting surface erosion and providing slope stability.

The introduction of geosynthetics in the late 20th century revolutionised slope reinforcement techniques. These lightweight, high-strength polymeric materials enhanced load distribution, facilitated drainage, and improved durability under both static and dynamic loading conditions[28,29]. Their versatility allowed engineers to design more flexible, efficient, and cost-effective systems compared to conventional solutions.

Pile systems, while used since antiquity for foundational stability, experienced major advances during the 20th century. Improvements in materials, design methodologies, and installation techniques enabled piles to provide reliable lateral and vertical support for unstable slopes. Their role expanded from simple foundations to integral elements in composite slope reinforcement strategies [30].

The emergence of geosynthetic-encased columns (GECs) and piled embankments marked significant milestones in this progression. GECs combine the load-bearing capacity of stone columns or piles with the lateral confinement provided by geosynthetic encasement, enhancing settlement control and slope stability. Cvetković et al. (2022)[31] demonstrated that this hybrid approach is particularly effective in soft or loose soils, where confinement prevents bulging and maintains structural performance. Large-scale tests further confirm the advantages of GECs in improving slope resilience under varied loading conditions [32].

Similarly, piled embankments improve slope stability by redistributing vertical loads and minimising settlement, especially in areas characterised by weak substrata or fluctuating groundwater levels [33,34]. By integrating piles with basal reinforcement or facings, these systems exemplify the move towards composite reinforcement solutions that maximise the synergistic benefits of different materials.

Overall, the historical progression of gabions, geosynthetics, and piles reflects a clear trajectory towards composite and hybrid systems. These innovations represent a paradigm shift in slope engineering, where material synergy and multi-mechanism reinforcement are harnessed to deliver sustainable, resilient, and cost-effective slope Stabilization strategies.

### Numerical and empirical modelling approaches

The evaluation of slope stability has long been underpinned by both empirical and numerical approaches, each offering unique strengths and limitations. Numerical modelling techniques, including the Limit Equilibrium Method (LEM), Finite Element Method (FEM), and Discrete Element Method (DEM), provide detailed simulations of soil–structure interaction. FEM in particular enables sophisticated analysis of stress–strain behaviour, pore water pressures, and load transfer under complex hydro-mechanical and dynamic conditions [33]. DEM and Material Point Methods (MPM) extend this capability by capturing granular soil behaviour and large-deformation processes, which are critical for modelling landslides and slope failures [28].

However, numerical models are highly dependent on accurate soil parameters, boundary conditions, and constitutive models. Critics highlight that this reliance can compromise predictive accuracy when field data are limited [35]. To address this, hybrid approaches combining numerical predictions with empirical field observations have gained momentum, ensuring validation of computational outputs against real-world performance.

Empirical methods, developed from decades of field case histories, remain widely used for their simplicity and practicality. These include stability charts, rock mass classifications, and regression-based predictive tools, which enable engineers to derive quick, conservative estimates of slope stability. Yet, empirical approaches often lack the fidelity to capture site-specific complexities, particularly under multi-hazard conditions such as rainfall-triggered and seismic-induced failures.

Current debates in the literature focus on reconciling these two paradigms. Many scholars argue for integrated frameworks that combine the precision of numerical modelling with the practicality of empirical design [29]. Such hybrid frameworks also align with optimisation techniques, including Artificial Intelligence (AI)-based predictive models, which can incorporate large datasets to refine design parameters and improve reliability.

In conclusion, numerical and empirical methods are complementary rather than mutually exclusive. While numerical approaches provide detailed mechanistic insights, empirical methods anchor designs in proven field evidence. Their integration offers a more balanced, reliable basis for slope stability analysis, particularly when applied to hybrid reinforcement systems that involve complex interactions between gabions, geosynthetics, and piles.

### Standardisation challenges

Despite significant progress in reinforcement technologies, challenges remain in the standardisation and codification of design practices for composite systems. Current design codes often treat gabions, geosynthetics, and piles as separate entities, without fully addressing their interactive behaviours when combined in hybrid configurations. This gap creates inconsistencies in safety factors, design assumptions, and performance evaluation across different regions and engineering practices [25].

Durability and long-term monitoring further complicate standardisation. Geosynthetics may experience creep, degradation under UV exposure, or clogging in drainage applications, while gabions are prone to corrosion of wire meshes in aggressive environments. Pile systems, meanwhile, face uncertainties related to soil–pile interaction under cyclic or seismic loading [18]. Without harmonised approaches, these uncertainties hinder the development of universally applicable design protocols.

Additionally, seismic design provisions for composite systems are limited. Current standards rarely incorporate probabilistic or performance-based methods that reflect real-world multi-hazard conditions. This restricts the ability of engineers to design resilient systems capable of withstanding both rainfall-induced and seismic instabilities [36].

Therefore, advancing slope Stabilization practice requires harmonised international codes that integrate hybrid behaviours, durability considerations, and multi-hazard performance. Rigorous testing, model validation, and international collaboration are critical to achieving standardised, reliable, and sustainable design guidance for composite reinforcement systems.

### Findings

#### Effectiveness of gabions and geosynthetics under rainfall conditions

Gabion walls are highly effective in controlling erosion and improving slope stability, particularly under rainfall infiltration scenarios. Their permeable structure enables drainage, which reduces hydrostatic pressures while dissipating the energy of surface runoff. This dual function contributes to enhanced stability and reduced risk of slope failures in rainfall-prone areas. Ferreira et al. (2020) [37] emphasized their ability to absorb and redistribute loads, findings that were corroborated by several field studies demonstrating the resilience of gabion walls under heavy rainfall [38].

The performance of geogrids and geotextiles is strongly influenced by three parameters: spacing, stiffness, and geometry. Closer spacing of reinforcement layers fosters effective load distribution and soil–reinforcement interaction, thereby increasing slope stability [39]. Stiffer reinforcement materials enhance load transfer efficiency and reduce deformation under applied stresses [40]. Geometry, including mesh size and shape, determines the tensile strength mobilized within the soil-reinforcement system and directly affects overall performance [41].

In comparisons between geocells, soil bags, and planar geosynthetics, geocells consistently outperform other systems by providing three-dimensional confinement of soil particles. This results in improved lateral restraint, increased shear strength, and enhanced load distribution, which is particularly advantageous for slopes subjected to high loads and severe erosion [42]. Soil bags, while effective as temporary stabilization measures and useful for rapid deployments, lack the durability and long-term effectiveness of geocells or planar reinforcements [43].

Case studies on municipal solid waste (MSW) slopes reinforced with geosynthetics highlight the unique challenges posed by heterogeneous and degradable materials. Reinforcement significantly improves the factor of safety by enhancing shear strength and controlling settlements. Ma & Javankhoshdel (2024) [44]noted that performance optimization requires careful consideration of waste variability, leachate effects, and site-specific load conditions. Shen et al. (2020) [45] further stressed the importance of long-term monitoring and adaptive management strategies, demonstrating that continuous data-driven reinforcement adjustments can sustain MSW slope stability over extended periods.

Collectively, these findings confirm that gabions, geogrids, geotextiles, and geocells provide distinct but complementary benefits for slope reinforcement under rainfall-induced stressors. Their performance is highly parameter-dependent, emphasizing the need for tailored designs to specific geotechnical and environmental conditions. The summary of research related to gabions and geosynthetics in rainy conditions is presented in Table 1.

#### Performance of geosynthetic-enclosed columns (GECs)

##### Effect of encasement stiffness and length on GEC-Supported slopes

The performance of geosynthetic-encased columns (GECs) in slope stabilization is highly dependent on the stiffness and length of the encasement. Increased encasement stiffness improves load-bearing capacity and reduces lateral displacement, thereby enhancing slope stability [[59]; [60]]. Longer encasements extend confinement and provide greater bulging resistance, particularly under dynamic loading, which further improves the stability of supported embankments [61].

##### Role of area-replacement ratio in optimizing GEC effectiveness

The area-replacement ratio, defined as the proportion of ground replaced by GECs, is a critical design parameter. Optimized ratios enhance load transmission, reduce differential settlement, and improve the composite stiffness of the reinforced ground. Maintaining ratios within recommended ranges ensures effective load-sharing and slope stability [62,63].

##### Influence of substratum consistency on settlement and displacement

The substratum consistency plays a vital role in governing settlement and lateral movement of GEC-supported systems. Softer substrata result in larger settlements and lateral deformations due to lower bearing capacity, while firmer substrata improve performance by limiting both [64,65]. This underscores the importance of detailed geotechnical site investigations prior to implementation.

##### Comparative advantages of GECs over unreinforced stone columns

Compared to traditional stone columns, **GECs** demonstrate superior performance. The geosynthetic encasement provides added confinement, which enhances lateral resistance and load-bearing capacity 60,66). GECs also promote faster drainage and reduce excess pore water pressures, which is critical in saturated soils [67]. Moreover, their resistance to bulging and settlement makes them better suited for high embankments and challenging site conditions [61,68].

In summary, GECs represent a significant advancement in slope stabilization, offering improved confinement, drainage, and load transfer capabilities compared to unreinforced stone columns. Their performance, however, is strongly influenced by encasement design parameters, area-replacement ratios, and substratum consistency, necessitating site-specific design adaptations. Several related studies are summarized in Table 2.

#### Performance of pile/column reinforcements

##### Influence of pile spacing, L/D ratio, and head fixity on slope stability

The stability of pile-reinforced slopes is significantly influenced by spacing, length-to-diameter (L/D) ratio, and head fixity. Closer spacing improves load transfer, enhances soil arching, and reduces lateral displacement risks [80,81]. Higher L/D ratios increase axial load-bearing capacity and improve slope resilience under dynamic loads ([82,83]. Head fixity is equally critical, as fixed-head piles provide greater lateral resistance and rotational restraint, substantially improving global stability [84].

##### Differences between end-bearing and floating piles

End-bearing piles stabilize slopes by transferring loads directly to competent strata, offering strong vertical resistance but potentially higher lateral displacements under seismic loading [85]. In contrast, floating piles distribute loads through shaft friction along their embedded length, which can be beneficial in soft soils but often results in larger lateral movements [86,87]. This fundamental difference in load-transfer mechanisms has major implications for pile design and application in varying soil conditions.

##### Performance under cyclic and seismic loading

Pile-reinforced slopes demonstrate notable resilience under cyclic and seismic conditions. Research shows that piles redistribute seismic forces and enhance slope stiffness, preventing catastrophic failures during earthquakes [88,89]. Wu et al. (2021) [90] and Zhang et al. (2020) [91] confirmed that piles mitigate lateral displacements and contribute to long-term stability. Nonetheless, cyclic degradation may reduce performance over time, highlighting the importance of robust design and ongoing monitoring [92].

##### Insights from centrifuge, numerical, and field studies

Experimental and numerical studies offer critical insights into soil–pile interaction. Centrifuge tests replicate real failure mechanisms, demonstrating how piles control deformation and delay slope failure. Numerical models capture soil-structure interaction and lateral load-sharing mechanisms, validating experimental outcomes [93]. Field studies further support these findings, documenting the long-term effectiveness of pile-reinforced slopes under diverse conditions [94,95]. Collectively, these studies underscore the importance of integrating laboratory, computational, and field data to optimise pile designs and configurations for slope Stabilization.

In conclusion, pile and column reinforcements are proven to enhance slope stability through optimal spacing, L/D ratios, and head fixity. Their performance under seismic and cyclic loads, as well as insights from multi-scale studies, reinforce their critical role in modern slope engineering. A summary of research on piles as reinforcement is presented in Table 3.

#### Hybrid and composite reinforcement systems

##### Performance of hybrid systems under multi-hazard conditions

Hybrid systems integrating gabions, geogrids, and piles demonstrate superior resilience in multi-hazard environments, including earthquakes, rainfall infiltration, and landslides. These systems leverage the strengths of each component—gabions provide erosion resistance and mass, geogrids offer tensile confinement, and piles contribute deep anchorage and load-bearing support. Field and numerical studies confirm that such configurations distribute stresses more evenly, reducing overall failure risks during simultaneous hazard scenarios [19].

##### Synergetic mechanisms from combining reinforcement systems

The combination of reinforcement methods produces synergetic effects, such as enhanced load sharing, increased lateral resistance, and improved drainage. For instance, gabion–geogrid interfaces minimise lateral displacements through better confinement, while piles transfer loads to deeper stable layers, reducing surface soil pressures. These interactions improve slope integrity, particularly under dynamic and hydro-mechanical stressors [19].

##### Settlement reductions and factor-of-safety improvements

Hybrid reinforcement systems also provide quantifiable improvements in settlement reduction and slope safety. Configurations that integrate geogrids and piles have been shown to reduce settlements significantly, sometimes by over 30 % while improving the factor of safety by >40 % compared to single-method systems [21,108]. The magnitude of these improvements depends on reinforcement arrangement, material type, and geometric design, underscoring the importance of site-specific optimisation.

##### Optimization techniques applied to hybrid systems

Recent advances incorporate computational optimisation tools such as Genetic Algorithms (GA), Particle Swarm Optimization (PSO), and Machine Learning (ML) to refine hybrid system designs. These methods enable multi-parameter optimisation—adjusting pile spacing, gabion dimensions, and geogrid properties—to achieve cost-effective, high-performance reinforcement. GA effectively navigates complex design spaces, while ML models predict outcomes based on historical data and adaptive learning [[109], [110], [111], [112]].

In summary, hybrid and composite systems offer robust, adaptable solutions for slope stability under multi-hazard conditions. Their synergetic mechanisms, settlement reduction benefits, and compatibility with optimisation tools position them as a critical component of next-generation geotechnical reinforcement strategies.

## Discussion

**Unified mechanistic framework** Hybrid systems mobilise complementary mechanisms: gabion drainage/mass, geogrid confinement/tension, and pile/column load transfer and arching. This section links the extracted parameter–response evidence to these mechanisms to support performance‑based design.. Numerical simulations that incorporate soil–structure interactions, drainage effects, and dynamic loading scenarios are essential to capture the complex synergy among these systems [120,121]. Such multi-faceted models can more accurately simulate static and dynamic conditions, offering engineers a robust framework for hybrid slope Stabilization.

### Cross-study triangulation: similarities, differences, and credibility

Across the body of evidence, several agreement zones emerge in which numerical, experimental, and field studies consistently reinforce one another—for example, demonstrating that increasing pile stiffness or reducing pile spacing enhances global stability, and that improved drainage capacity reduces pore-pressure accumulation and consequently increases the factor of safety. At the same time, notable disagreement zones persist, particularly where 2D numerical models tend to overpredict stability improvements compared with 3D analyses, or where simplified drainage assumptions lead to underestimation of transient pore-pressure rise during infiltration or cyclic loading. To ensure a balanced synthesis, the review applies explicit credibility checks, assigning greater evidential weight to studies that document validation, mesh-convergence procedures, and sensitivity analyses, while treating unvalidated parametric simulations as exploratory or hypothesis-generating rather than confirmatory.

### Advanced numerical modelling under coupled rainfall–seismic loading

This subsection synthesizes how the reviewed numerical studies treat soil–structure interaction (SSI) and multi-field coupling by outlining key modelling practices and identifying minimum reporting requirements, noting that SSI is commonly represented using interface elements, contact formulations, or embedded pile techniques, although validation against 3D finite-element or centrifuge/field data remains limited—hence the recommendation for 3D benchmarking, including insights such as those provided by Asgari et al. (2024) [131] for saturated and dry deposits. In terms of hydro-mechanical coupling, fully coupled seepage–deformation analyses are preferable for conditions involving rapid pore-pressure evolution (e.g., rainfall or cyclic loading), whereas staged or quasi-static approaches tend to underpredict transient pore pressures and misestimate the factor of safety. Dynamic modelling should clearly report the damping model employed (Rayleigh or hysteretic), the target frequencies, and acceleration-scaling parameters, as frequency-dependent damping influences deformation patterns and cyclic pore-pressure buildup. Variability in gabion–pile interaction modelling—some studies using explicit numerical contact–stiffness representations while others infer mechanisms experimentally—highlights the need for integrated 3D modelling supported by instrumented validation. Mesh convergence and geogrid strain accuracy also require explicit documentation, as refinement near interfaces and checks for strain localization are often omitted. When hysteretic models such as Bouc–Wen are applied, key parameters governing stiffness degradation and energy dissipation (α, β, γ, n) should be transparently reported alongside calibration targets. Likewise, rainfall-induced infiltration studies should specify boundary conditions and clarify whether soil–water characteristic curves (SWCC) are included, as omission can distort suction and unsaturated-strength behaviour. For saturated or liquefiable soils, two-phase Biot formulations or equivalent effective-stress models are recommended since single-phase approximations may fail to capture coupled pore-pressure–displacement responses, and the limited consideration of post-earthquake pore-pressure redistribution restricts long-term settlement predictions. The cyclic behaviour of geosynthetic-encased columns similarly depends on encasement stiffness and length, yet 3D cyclic analyses remain scarce—though recent multi-hazard dynamic simulations, such as those in Sorkhi, et al. (2025)[132], illustrate emerging practices. Comparisons between end-bearing and floating piles under cyclic loading also remain incomplete, particularly regarding L/D ratios, arching mechanisms, and lateral-spreading resistance, warranting further insights from works such as Bagheri et al. (2025)[133] and Asgari et al. (2025) [134]. Finally, multidirectional earthquake inputs and higher-mode SSI effects are seldom explored within hybrid gabion–pile systems, making this a critical area for future modelling research.

### Optimisation and AI: predictive vs calibration role

Machine-learning (ML) frameworks in the reviewed studies serve two distinct yet complementary roles: first, as predictive modelling tools capable of estimating key performance indicators—such as the factor of safety and settlement—based on input soil and reinforcement parameters, effectively functioning as surrogate models that reduce dependence on computationally expensive finite-element simulations; and second, as design-calibration and decision-support instruments, where ML or optimisation algorithms are employed to refine design parameters so that they satisfy specific performance targets, engineering constraints, and constructability requirements, thereby enhancing both efficiency and reliability in the design process.

### Beyond deterministic optimisation: reliability-based design optimisation (RBDO)

While deterministic optimisation approaches such as GA and PSO can efficiently identify parameter combinations that improve slope performance, they do not explicitly account for soil variability or modelling uncertainty, which can lead to overconfident or non-robust design recommendations. In contrast, reliability-based design optimisation (RBDO) incorporates failure probability directly into the optimisation process, enabling a balanced evaluation of cost, performance, and safety under inherent geotechnical uncertainties. As an example of emerging practice, Hu et al. (2023)[135] demonstrated a first-order RBDO framework with Pareto optimality for 3D pile-reinforced slopes, highlighting the potential of RBDO to provide more resilient and uncertainty-aware design guidance for hybrid reinforcement systems.

### Non-homogeneous slopes: advanced analytical methods

A significant limitation identified across many numerical studies is the pervasive reliance on the uniform soil assumption, which oversimplifies natural stratigraphy and can lead to biased predictions of stability and deformation. More advanced analytical frameworks—such as the Discrete Kinematic Mechanism (DKM) approach—offer a powerful alternative by enabling rigorous stability evaluations for non-homogeneous slopes, with the capability to incorporate 3D geometries, seismic loading, and pore-pressure effects. This methodological advancement is well illustrated in the work of Sun et al. (2018) [136], which provides a foundational DKM formulation that can substantially enhance the realism and reliability of future hybrid system analyses.

### Design code gaps across regions

To contextualize the practical implications of the reviewed findings, it is essential to examine how existing regional design standards address—or fail to address—the requirements of hybrid reinforced systems. Despite substantial advances in numerical modelling and experimental insights, current codes remain fragmented in their treatment of multi-element reinforcement, soil–structure interaction, and coupled hydro-mechanical behaviour. The following table summarises key gaps across major international and national standards, highlighting areas where further refinement or guidance is needed to support consistent and reliability-based design practice for hybrid gabion–geogrid–pile systems (Table 5).

**Table 5:** Indicative code gaps for hybrid systems.

Code/guideline	Typical coverage	Gap for hybrid gabion–geogrid–pile/GEC systems	Practical implication
Eurocode (e.g., EC7/EC8)	Geotechnical design + seismic	Limited explicit guidance for combined systems; coupling/SSI not operationalised	Engineers rely on project–specific modelling/validation
ASTM / FHWA manuals	Materials / highway geotech	Component‑level guidance dominates; hybrid interaction effects not codified	Conservative designs or inconsistent assumptions
JGS / Japanese guidelines	Advanced seismic geotech	Strong seismic focus but hybrid composite detailing varies	Need harmonisation for gabion‑faced composite systems
Indonesian standards	Local practice	Often limited by local datasets for calibration	Higher uncertainty; monitoring and validation critical

### Sustainability, LCCA, constructability, and “green–grey” durability

A comprehensive sustainability assessment of hybrid reinforcement systems requires integrating life-cycle cost analysis (LCCA) to ensure that observed performance gains are weighed against installation complexity, equipment demands, time requirements, and the associated QA/QC costs. From a carbon and environmental perspective, hybrid configurations may indeed reduce reliance on high-carbon structural materials; however, such benefits must be balanced against the embodied energy, transportation footprint, and long-term durability of geosynthetics. Moreover, comparisons between green and grey reinforcement strategies highlight the need to evaluate long-term degradation mechanisms—particularly for bio-based, vegetative, or bamboo-grid solutions—which may respond differently than geosynthetics when subjected to coupled hydro-mechanical stresses, chemical exposure, and environmental weathering over the service life of the system.

### Standardised long‑term monitoring protocols

To address the persistent issue of sparse and inconsistent field monitoring, we recommend establishing a minimum monitoring protocol that defines a core set of key performance indicators (KPIs) essential for reliable evaluation of hybrid reinforcement systems. These KPIs should include pore-pressure measurements at soil–soil-reinforcement interfaces and within geosynthetic-encased or stone columns; geogrid strain distributions and connection loads; bending-moment and shear-force profiles along piles; surface settlement and lateral displacement obtained from inclinometers; as well as rainfall intensity, infiltration boundary conditions, and groundwater-level observations. Collectively, these metrics provide a coherent basis for developing comparable monitoring databases that can support more rigorous model calibration, reliability-based design optimisation (RBDO), and future progress in design code development.

### Comparative strengths and weaknesses of reinforcement methods

Each reinforcement method presents specific advantages and limitations. Gabions excel in erosion control and providing structural mass, but may degrade physically over time. Geosynthetics offer tensile reinforcement and drainage efficiency, though their effectiveness is sensitive to installation quality and environmental degradation. Piles provide strong vertical and lateral support but are associated with higher costs and technical challenges during installation. Hybrid systems, by leveraging the strengths of each method, deliver improved overall performance but also introduce design and construction complexities requiring advanced engineering expertise [111].

### Settlement reductions and factor-of-safety improvements in hybrid configurations

Settlement reductions and factor-of-safety (FOS) improvements vary depending on hybrid system configurations. Dense gabion structures combined with geogrid layers have been shown to reduce settlements and improve safety factors more effectively than single-method solutions [111]. Incorporating piles into these systems further enhances load distribution and stability, particularly under seismic conditions, delivering substantial improvements in both settlement control and FOS [120]. These findings highlight the importance of design optimisation tailored to site-specific geotechnical conditions.

### Optimisation techniques for hybrid slope reinforcement systems

Advanced optimisation techniques are increasingly applied to hybrid reinforcement systems to maximise performance and cost efficiency. Genetic Algorithms (GA) are effective in exploring large design spaces, Particle Swarm Optimization (PSO) fine-tunes parameters through swarm intelligence, and Machine Learning (ML) enables predictive modelling and adaptive design based on performance data [16,111]. Together, these approaches help engineers balance material usage, construction costs, and long-term stability outcomes.

### Methodological limitations in current research

Current research faces several methodological challenges. Scale effects remain a significant limitation, as behaviours observed in laboratory-scale models may not accurately replicate field-scale performance [122,123]. Monitoring gaps persist, with limited long-term field data available to validate numerical predictions, leading to uncertainty in real-world applications [124,125]. Furthermore, modelling assumptions such as uniform soil properties or simplified boundary conditions often fail to capture the complexity of natural systems, reducing model reliability [126,127].

### Implications for design codes, sustainability, and engineering practice

The increasing adoption of hybrid reinforcement systems has important implications for design codes, sustainability, and engineering practice. Current design codes require revision to account for the unique behaviours of composite systems, particularly under multi-hazard conditions [123,127]. From a sustainability standpoint, composite systems reduce material use by exploiting the complementary strengths of different reinforcements, lowering the environmental footprint of infrastructure projects (RF-SVR Model, 2024). Engineering practice is also evolving with the integration of novel materials, AI-enhanced modelling, and optimisation tools, which enable more efficient designs and better predictions of slope behaviour [128,129].

In summary, hybrid and composite systems represent the future of slope Stabilization by combining diverse reinforcement mechanisms into resilient, sustainable, and cost-effective solutions. However, advancing their application requires improvements in modelling accuracy, long-term monitoring, and harmonisation of design standards to ensure global applicability and safety.

## Conclusion

Composite reinforcement systems combining gabions, geogrids, and piles/columns improve stability and deformation control under multi‑hazard conditions by mobilising drainage, confinement, arching, and deep load transfer. Evidence synthesis indicates that hybrid configurations can achieve substantial FoS gains (up to ∼45 %) and settlement reductions (>30 %), but these benefits depend strongly on reinforcement stiffness/geometry, pile spacing and head fixity, drainage controls, and pore‑pressure evolution under coupled rainfall–seismic actions. Methodological gaps remain: many numerical studies lack 3D validation, explicit reporting of coupling assumptions, damping formulations, and mesh convergence; long‑term field monitoring is still sparse, limiting generalisable design guidance. To advance the field, future research should prioritise (i) validated 3D coupled hydro‑mechanical‑dynamic modelling with transparent reporting, (ii) uncertainty‑aware optimisation via RBDO, (iii) non‑homogeneous slope frameworks, and (iv) harmonised design‑code provisions supported by shared datasets. Finally, sustainability should be operationalised through LCCA and carbon‑aware comparisons of green–grey reinforcement options, supported by standardised monitoring protocols that generate comparable long‑term performance databases.

## Ethics statements

The author states that all writing, data and tables in this paper do not come from social media platforms.

## CRediT author statement

Devi Oktaviana Latif: Writing – review & editing, Methodology, Conceptualization. Virananda Samudera Rahmadhian: Writing, editing, Data curation. Amalia Ula Hazhiyah: Writing – review & editing, Data curation. '

## Declaration of competing interest

The authors declare that they have no known competing financial interests or personal relationships that could have appeared to influence the work reported in this paper.

## Data Availability

The authors are unable or have chosen not to specify which data has been used.

## Appendix Supplementary materials

Image, application 1

Image, application 2
