# Supplementary material for: Systematic review of gabion-faced geogrid and pile systems for slope and embankment stability
Source: MethodsX. 2025 Dec 17;16:103767. doi: 10.1016/j.mex.2025.103767 (PMC12808513; doi:10.1016/j.mex.2025.103767)
Supplement: Supplementary file 1 [file mmc1.docx]

# Supplementary Material

Article Title:
Systematic Review of Gabion-Faced Geogrid and Pile Systems for Slope and Embankment Stability

Journal: MethodsX
Reference: MEX 103767

## Table S1. PRISMA Screening and Selection Summary

This table documents the PRISMA-guided screening process applied in this systematic review.

- Records identified through Scopus database searching: 652
- Records excluded based on publication year (before 2020): 353
- Records excluded after title/abstract screening (out of scope): 170
- Full-text articles assessed for eligibility: 129
- Studies included in qualitative synthesis: 66

## Table S2. Data Extraction Codebook

For each included study, the following metadata were extracted:

1. Bibliographic information (author, year, country)
2. Study type (field, centrifuge, laboratory, numerical, analytical)
3. Soil condition (soil type, saturation state, groundwater condition)
4. Reinforcement configuration (gabion geometry, geogrid stiffness/spacing, pile or GEC dimensions)
5. Loading conditions (static, rainfall infiltration, seismic/dynamic loading)
6. Modelling approach (2D/3D FEM, HM coupling, SSI treatment)
7. Key performance outcomes (FoS, settlement, displacement, pore pressure)

## Table S3. List of Included Studies (Summary)

A total of 66 peer-reviewed studies published between 2020 and 2025 were included.
These studies cover gabion-faced geogrid walls, pile-supported slopes, geosynthetic-encased columns (GECs), and hybrid reinforcement systems under static, rainfall-induced, and seismic loading conditions.

## Table S4. Reproducibility and Data Availability Notes

This supplementary material is provided to enhance transparency and reproducibility. All screening decisions, inclusion criteria, and extracted parameters follow the PRISMA 2020 framework. The compiled dataset can be reused for comparative studies, optimisation frameworks, and reliability-based design investigations.
